# Determining the chemical exchange saturation transfer (CEST) behavior of citrate and spermine under in vivo conditions

**DOI:** 10.1002/mrm.25997

**Published:** 2015-10-15

**Authors:** Meer Basharat, Nandita M. deSouza, Harold G. Parkes, Geoffrey S. Payne

**Affiliations:** ^1^ CRUK Cancer Imaging CentreInstitute of Cancer Research and Royal Marsden NHS Foundation TrustDowns RoadSuttonSurreyUnited Kingdom

**Keywords:** CEST, spermine, citrate, exchange, QUEST

## Abstract

**Purpose:**

To estimate the exchange rates of labile ^1^H in citrate and spermine, metabolites present in prostatic secretions, to predict the size of the citrate and spermine CEST effects in vivo.

**Methods:**

CEST z‐spectra were acquired at high‐field [11.7 Tesla (T)] from citrate and spermine solutions at physiological pH (6.5) using saturation power 6 μT. CEST was performed at different temperatures to determine exchange regimes (slow, intermediate or fast). For low pH solutions of spermine, exchange rates were estimated from resonance line width, fitting z‐spectra using the Bloch equations incorporating exchange, and using quantifying exchange using saturation time experiments (QUEST). These rates were extrapolated to physiological pH.

**Results:**

Citrate showed little CEST effect at pH 6.5 and temperature (T) = 310 K (maximum 0.001% mM^‐1^), indicating fast exchange, whereas spermine showed greater CEST effects (maximum 0.2% mM^‐1^) indicating intermediate‐to‐fast exchange. Extrapolating data acquired from low pH spermine solutions predicts exchange rates at pH 6.5 and T of 310 K of at least 2 × 10^4^s^‐1^.

**Conclusion:**

Citrate and spermine show minimal CEST effects at 11.7T even using high saturation power. These effects would be much less than 2% at clinical field‐strengths due to relatively faster exchange and would be masked by CEST from proteins. Magn Reson Med 76:742–746, 2016. © 2015 The Authors. Magnetic Resonance in Medicine published by Wiley Periodicals, Inc. on behalf of International Society for Magnetic Resonance in Medicine. This is an open access article under the terms of the Creative Commons Attribution License, which permits use, distribution and reproduction in any medium, provided the original work is properly cited.

## INTRODUCTION

MRI examinations typically create contrast between tissues in the body by exploiting the different NMR relaxation times of water ^1^H nuclei. An alternative contrast mechanism is chemical exchange saturation transfer (CEST), which interrogates ^1^H nuclei in molecules such as metabolites and proteins (pool **s**), which are exchanging with ^1^H nuclei in water (pool **w**). CEST contrast is produced by applying a saturation pulse at the resonance frequency of the ^1^H in pool **s** (ν_s_). The saturated ^1^H nuclei in pool **s** then exchange with unsaturated ^1^H nuclei in water at rate k_sw_. After successive saturation‐and‐exchange events the water signal is attenuated.

CEST contrast is advantageous for measuring low‐concentration ^1^H pools because the water signal attenuation may be tens or hundreds fold larger than the inherent MR signal from these nuclei. CEST has been applied to image the distributions of myo‐inositol 1, creatine 2 and glutamate 3 in humans. In the prostate, CEST effects have previously been attributed to the ^1^H nuclei of protein amides 4. However, the glandular metabolites citrate and spermine (normal concentrations of 40 mM and 10 mM 5, and containing labile ‐OH and –NH_x_ groups, respectively, see Supporting Figure S1, which is available online) are large potential CEST sources in the prostate. While the concentrations of these metabolites decrease significantly from normal in benign and malignant prostate disease 6, it is not always easy to visualize them using MR spectroscopy due to spectral overlap and lipid contamination. Because characterization, evaluation, and optimization of the CEST effects of citrate and spermine have not yet been reported, in this study the exchange conditions and estimates of the chemical exchange rates of ^1^H in citrate and spermine with water were determined. This then provides predictions for CEST behaviors at physiological pH and temperature and relevant field‐strengths.

## METHODS

### Theoretical Background

The amount of CEST that occurs from a ^1^H species depends on its concentration, chemical exchange rate with ^1^H in water and the relaxation rates. Very slow exchange causes little CEST. CEST is also diminished at high exchange rates at which the resonances of exchanging ^1^H species coalesce because the CEST saturation pulse is unable to selectively saturate pool **s** without some direct saturation of pool **w**. The chemical exchange rate for coalescence is given by 7

(1) $$k_{\text{sw}}=2^{-½}\pi \Delta \nu \approx 2.22\Delta \nu$$

where Δν is the chemical shift difference, ν_s_ ‐ ν_w_. The greatest CEST effects, therefore, occur for ^1^H with intermediate exchange rates, k_sw_ < 2.22Δν 8. For example, at field‐strength 7 Tesla (T) the ^1^H exchange rate in glutamate [k_sw_ = 875s^‐1^ 3] is nearly optimal for CEST (at 7T, Δν = 894s^‐1^, so k_sw_ = 0.98Δν).

Methods to measure the chemical exchange rates of slowly‐exchanging ^1^H include measurement of the resonance linewidth 9, fitting the Z spectra of the full Bloch equations including exchange 10, and using the method of quantifying exchange using saturation time (QUEST, see below) 9. Exchange rates at different pH values can be estimated using 11;

(2) $$k_{\text{sw}}\left(\text{pH}\right)={\text{k}}_{0}+{\text{k}}_{a}10^{\left(-\text{pH}\right)}+{\text{k}}_{b}10^{\left(\text{pH}-\text{pKw}\right)}$$

where k_0_ is the spontaneous exchange rate, k_a_ is the acid‐catalyzed exchange constant, k_b_ is the base‐catalyzed exchange constant, and pK_w_ is the water ionization constant [13.62 at 310 K 12]. k_sw_ has a minimum value at pH = ½(log_10_(k_a_/k_b_)+pK_w_), which commonly occurs at pH 3–4 13, and increases for all other pH values.

### Quantifying Exchange Using Saturation Time

Under many conditions, the asymmetric magnetization transfer ratio (MTR_asym_) can be shown to have the form 9:

(3) $${\text{MTR}}_{\text{asym}}=(S_{w}(-\Delta )\;–{\text{ S}}_{w}(+\Delta ))\;/S_{0w}=\text{ p}\left(1-e^{-\text{qt}}\right)$$

where p and q are constants, t is the saturation pulse length and S_w_(Δ) is the attenuated water signal due to saturation at frequency offset Δ. Parameter q is dependent on k_sw_, the water relaxation rate (R_1w_) and the relative numbers of ^1^H in pool **i** involved in exchange at irradiation frequency offset Δ 9;

(4) $$k_{\text{sw}}(\Delta )=(q(\Delta )-{\text{R}}_{\text{1w}})*(n_{w}/n_{s}(\Delta )).$$

Exchange must be relatively slow. In the QUEST method 9 S_w_(Δ) is measured at a range of saturation durations, t, with Δ set to the frequency of the exchanging hydrogens (and to the corresponding frequency on the other side of water). Fitting Eq. (3) to the data yields parameter q; k_sw_ is obtained using Eq. (4).

### Experimental Procedures

Spectra were acquired using a 5 mm BBO probe in a 500 MHz vertical bore system (Bruker) to exploit the high values of Δν and so increase the signal change from the CEST effect. Chemical shift offsets are given relative to water unless otherwise stated.

#### Resonance Offset Determination

A total of 100 mM citrate was scanned at 310 and 277 K, pH 6.5 and 2.0, with 64 repetitions and 16k complex points over 10 kHz bandwidth. The 20 mM spermine was scanned at 301 K, pH 3.1 and 4.3, with 128 repetitions and 16k complex points over 6 kHz bandwidth.

#### Exchange Regime Determination

To determine the exchange regime (slow, intermediate or fast), the relationship of chemical exchange rate with temperature was exploited. At constant pH, k_sw_ increases with increasing temperature (Eyring equation). Increased k_sw_ produces more CEST if k_sw_ is slow at the lower temperature, but reduced CEST if already in intermediate exchange and the coalescence condition is approached.

Solutions of 100 mM citrate and 20 mM spermine tetrahydrochloride were studied at physiological pH [6.5 14] using sodium hydroxide to achieve the required pH. CEST effects were measured using a saturation time of 1 s and temperatures of 280 K, 295 K and 310 K. A total of 100 mM citrate was also investigated at pH 2.0, 310 K, and with 4 s saturation. Acquisition parameters included 16 repetitions, 20 s repetition time, 16 k complex points, and 6 kHz bandwidth. Saturation was achieved using a continuous‐wave block saturation pulse with amplitude 6 μT (255 Hz). The saturation pulse amplitude was verified by measuring the pulse length required for a 90‐degree pulse. S_w_(Δ) was measured by integrating the absorption signal from +0.5 to ‐0.5 ppm relative to water. The attenuation due only to CEST was determined by cancelling out the symmetric direct saturation component of each z‐spectrum by calculating the asymmetric magnetization transfer ratio (Eq. (3)).

Average MTR_asym_ was calculated between +1.2 ppm to 0 ppm for citrate (hydroxyl groups expected at approximately +0.8 to +0.6 ppm), and from +5.0 ppm to 0 ppm for spermine (amine groups expected at +3 to +4 ppm). Average MTR_asym_ is analogous to the MTR_asym_ integral used in others studies 15, 16 but more intuitively describes the amount of CEST occurring.

#### Exchange Rate Measurements on Spermine

Exchange rates were estimated from solute peak linewidths, fitting z‐spectra to the full Bloch equations using exchange, and using the QUEST method. Spermine solutions at pH 3.1 and 4.3 were measured. These were lower than that of prostatic fluid [pH approximately 6.5 14] with the intention of achieving slower exchange rates that are more readily measured.

Linewidth measurements were made using pulse‐acquire spectra without water suppression (repetition time of 5.7s, 128 repetitions, other parameters as above). The exchange rate was calculated using 9;

(5) $$k_{\text{sw}}=\pi .{\text{LW}}_{s}–{\text{R}}_{\text{2s}}.$$

Z‐spectra were acquired by measuring the amplitude of the bulk water signal, S_w_(Δ), after applying saturation at offset frequencies Δ from +5.0 to ‐5.0 ppm relative to water, in 0.2 ppm increments. The saturation duration was 4 s. S_0w_ was found by performing the measurement using a saturation pulse with offset frequency Δ = +20 ppm. Resulting z spectra were fit using the full Bloch equations including exchange [based on 10 but modified to run in IDL]. B_1_ and saturation times were fixed at the values specified above, while the chemical shift offsets, relaxation parameters and exchange rate were fitted to the data. To reduce the number of free parameters, it was assumed that both amine pools had the same values of T_1_, T_2_, and exchange life time.

For QUEST measurements, water spectra were acquired with saturation durations of 0, 0.5, 1.0, 2.0, 4.0, 8.0 and 12.0 s at frequency offsets of ±3.0 ppm relative to water, corresponding to the position of the main resonance peak observed in the pulse‐acquire spectra. Plots of MTR_asym_ versus saturation time t were fitted in IDL to find values for p and q using Eq. (3).

R_1w_ was measured by performing inversion‐recovery experiments using inversion times, TI, of 1 s to 20 s in 1 s increments, and recovery delay of 15s. Data were fitted using

(6) $$S_{w}{/S}_{\text{0w}}=1-a{\text{.e}}^{(-R_{\text{1w}}\text{.TI)}}.$$

A 3‐mm tube in the 5‐mm probe was used to minimize the effects of radiation damping.

#### Estimation of Exchange Rates at pH 6.5

Exchange rates were extrapolated to pH 6.5 by using k_sw_(pH 3.1) and k_sw_ (pH 4.3) to determine k_0_ and k_b_ in Eq. (2). The k_a_ term was omitted because only chemical exchange rates for pH > 3 were considered.

## RESULTS

### Citrate

No peaks were visible in pulse‐acquire spectra of citrate at pH 6.5 and at pH 2.0 except for that from water, even at 277 K. This suggests either fast exchange, or a hydroxyl peak that is within the linewidth of the water resonance. Simulations based on standard exchange lineshape analysis [e.g., Gunther 17] show that a separate 100 mM resonance at 1 ppm offset, intrinsic linewidth 1 Hz, B_0_ = 11.7T, broadens to invisibility for k_sw_ larger than ∼2000 Hz.

A total of 100 mM citrate solution at pH 6.5 and temperature (T) = 310 K, 295 K, and 280 K demonstrated negligible CEST, with average MTR_asym_ of 0.1% < 0.01%, and < 0.01%, respectively. All these measurements of CEST in citrate are effectively zero, with no significant difference between them. This is consistent with chemical exchange in citrate being in the fast exchange regime at 11.7T and pH 6.5. Likewise, no CEST effects were observed for 100 mM citrate at pH 2.0 and T = 310 K, where the exchange rates should have been substantially lower than at pH 6.5 and the same temperature. Because no peaks or CEST effect were observed at any temperature or pH measured, the determination of k_sw_ was not attempted.

### Spermine

**Figure 1:**
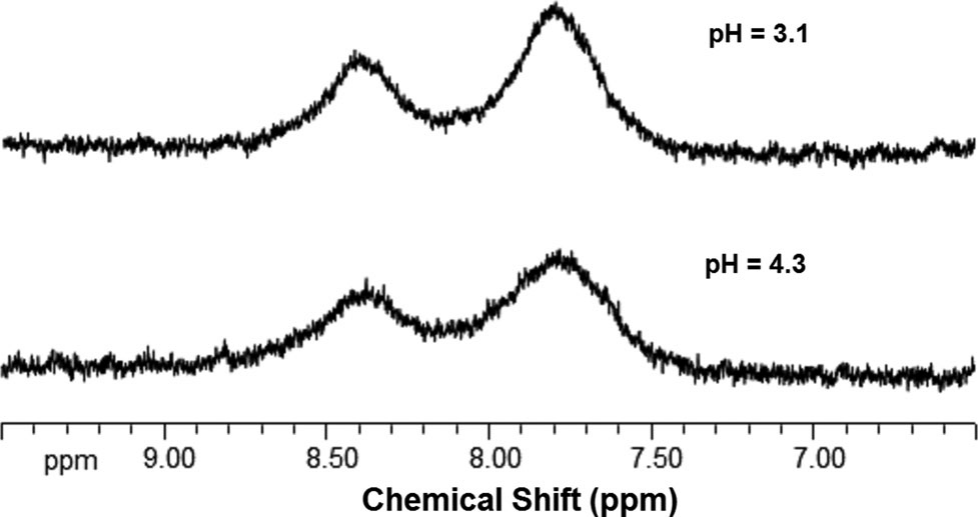
Pulse‐acquire ^1^H NMR spectra of spermine at pH 3.1 and 4.3, showing the chemical shift offsets of the amine resonances (relative to TMS; peak positions relative to water are approximately 3.0 and 3.7 ppm). Acquisition parameters included temperature = 310 K, spectral width 6009 Hz, 16 k complex points, TR = 5.73 s, 128 repetitions.

Pulse‐acquire spectra of 20 mM spermine at 310 K and pH 3.1 and 4.3 are shown in Figure 1. The amine resonances are seen at approximately 3.0 and 3.7 ppm relative to water (giving exchange coalescence limits of approximately 3300 and 4100 Hz, respectively). At pH 4.3, the linewidths are 187 Hz and 177 Hz, respectively. Given that R_2s_ is expected to be similar to that of the water (∼ 1 s^‐1^) the linewidth is dominated by the exchange rate, which using Eq. (5) can be estimated as approximately 590 Hz and 570 Hz, respectively. At pH 3.1, the corresponding estimated exchange rates are 470 Hz and 440 Hz, respectively. The water line widths in these samples were in the range 9.5 to 12.5 Hz.

**Figure 2:**
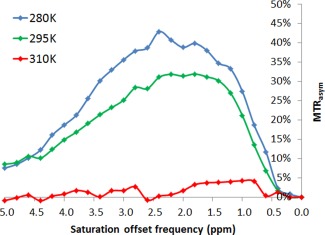
MTR_asym_ plots from z‐spectra acquired from 20 mM solutions of spermine at pH 6.5. The increasing CEST effect at lower temperature (reducing k_sw_) indicates chemical exchange in the intermediate‐to‐fast regime at T = 310 K

The 20 mM spermine at pH 6.5 and T = 310 K demonstrated a broad CEST effect with average MTR_asym_ = 1.36% (Fig. 2). The amount of CEST from spermine at pH 6.5 increased with decrease in temperature, with average MTR_asym_ = 18.7% at T = 295 K and average MTR_asym_ = 24.0% at T = 280 K. This increase in CEST with reducing temperature (and hence exchange rate) indicates that chemical exchange in spermine is in the intermediate‐to‐fast regime at pH 6.5 and field‐strength 11.7T.

**Figure 3:**
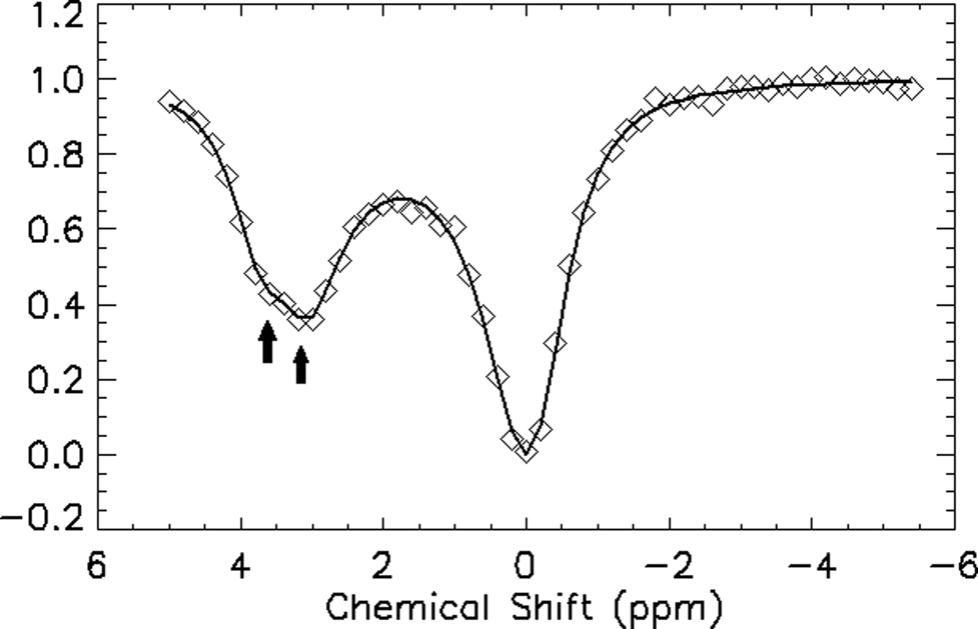
Z‐spectrum obtained from 20 mM spermine at pH 4.3 and at 310 K. Water signals at each time point were acquired with spectral width 6009 Hz, 16 k complex points, repetition time = 22.73 s, and 4 repetitions. The fitted curve was obtained using a 3‐pool CEST fitting routine described in Woessner et al 10 but modified for use in IDL.

A full z‐spectrum of spermine at pH 4.3 and 310 K using saturation pulses of duration 4 s is shown in Figure 3. The resulting calculated exchange rates were 461 Hz (pH 3.1) and 676 Hz (pH 4.3).

**Figure 4:**
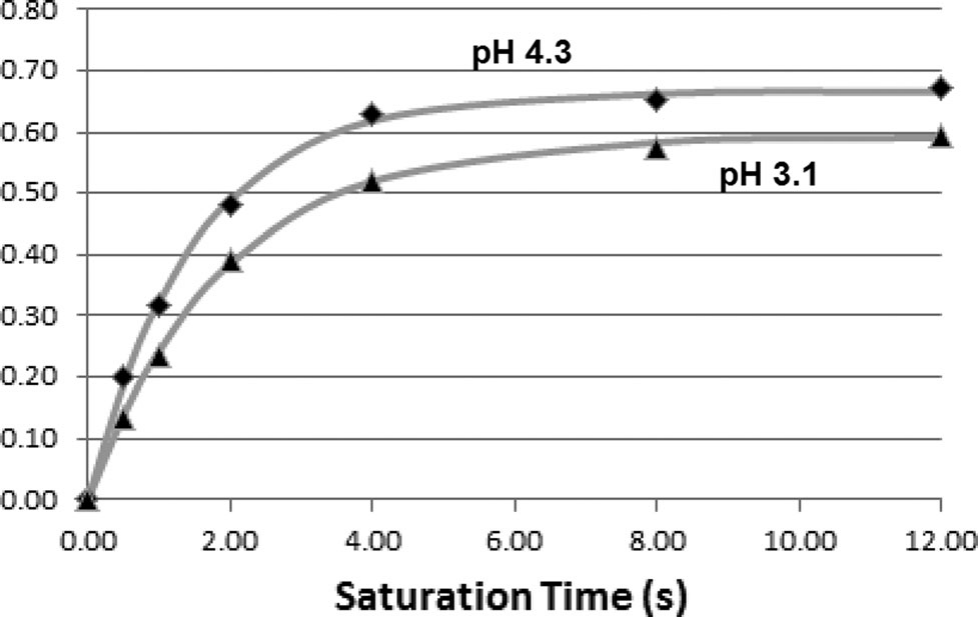
Quantifying Exchange using Saturation Time (QUEST) fits of spermine CEST at Δ= +3.0 ppm, pH values of 3.1 and 4.3, and at 310 K. Due to base‐catalyzed chemical exchange, exchange and CEST are greater at pH 4.3 than at pH 3.1.

The chemical exchange rates for spermine at an offset frequency of 3.0 relative to water were calculated using QUEST at pH 3.1 and 4.3, with T = 310 K. The fitted curves are shown in Figure 4. Equation (6) yielded R_1w_ as 0.207 s^‐1^ and 0.208 s^‐1^ at pH 3.1 and 4.3, respectively (and “a” as 1.99). With fitted q values of 0.525 and 0.661, and a ratio (n_w_/n_s_) of 110000/120 (i.e., assuming 6 hydrogen nuclei involved in exchange in 20 mM spermine at this offset) this results in k_sw_ of 291 and 416 Hz, respectively.

The exchange rates measured using the three different methods are summarized in Table 1, together with the predicted exchange rates at pH 6.5 obtained by extrapolation.

**Table 1:** Summary of Measured Spermine Exchange Rates (pH 3.1 and 4.3), with Extrapolation to pH 6.5^a^

	k_sw_ (Hz)			Fitted k_b_	Fitted k_0_
Method	pH 3.1	pH 4.3	pH 6.5 (extrapolated)	(Hz)	(Hz)
Linewidth	453	572	20,580	2.65 x10^11^	445
Fit to z spectrum	461	676	36,820	4.79 x10^11^	447
QUEST	291	416	21,430	2.79 x10^11^	283

a The k_sw_ values calculated from the linewidth measurements were averaged over the two peaks.

## DISCUSSION

In this study, no resonance peaks or CEST effects were observed for citrate, even when pH was reduced to 2.0 and temperature to 277 K This is consistent with fast exchange. It is also possible that the resonance peak was so close to water that no peaks could be observed.

For spermine, by decreasing the pH the exchange rate was measured in the slow exchanging regime, and then extrapolated to physiologic pH and faster exchange regimes, using the known k_sw_(pH) relationship. Results indicated that chemical exchange in spermine is in the intermediate‐to‐fast exchange regime at 11.7T, for pH 6.5 and temperature of 310 K, with an extrapolated exchange rate of at least 2 × 10^4^ s^‐1^ (Table 1). This is roughly 10 times faster than chemical exchange rates observed in creatine [950 s^‐1^ 2] and glutamate [875s^‐1^ 3], and well above the coalescence limit (3300 and 4100 Hz for the 3.0 and 3.7ppm peaks, respectively). Such rapid exchange is consistent with the small CEST profile observed for spermine at 310 K (Fig. 2).

In consequence of their rapid exchange rates, neither citrate nor spermine will be likely to contribute to CEST effects in the prostate at clinical field‐strengths, as at lower fields the rate of exchange relative to the chemical shift difference between the ^1^H nuclei in pools **s** and **w**, will be even larger, with greater coalescence of peaks. CEST effects from exchanging nuclei in other molecules such as proteins are, therefore, predicted to completely conceal the small CEST effects from citrate and spermine in the prostate in vivo.

Conditions which affect the CEST effect from metabolites include saturation power and duration, tissue pH, and relaxation times. A lower saturation power than used in this study (6 μT) would probably reduce the CEST effect but may also reduce direct saturation of the water. The pH of the prostate does vary, but pH 6.5 is at the acidic end of the measured physiological range for prostatic fluid: 6.2–8.0 14, 6.7 18, and 6.6 19; more alkaline pH will elicit faster chemical exchange and even less CEST. While the difference in relaxation characteristics in vivo compared with our solutions will have some effect on the amount of CEST signal anticipated, spermine and citrate are primarily located in the prostatic ducts and are, therefore, relatively mobile with expected relaxation times not greatly different to those in solution.

The three different methods for measuring spermine exchange rates produced values of a similar magnitude but with the QUEST method producing much lower values. The reason for this may be related to the QUEST calculation assuming instantaneous equilibrium 9, a condition which will not be quite valid for metabolites in dilute solution for which T_2s_ is expected to be relatively long. The small uncompensated direct saturation of water may also contribute, as the water linewidths (approximately 10 Hz) suggest that R_2w_ is larger than the 6 Hz limit suggested in 9 to not require correction. However, the use of MTR_asym_ rather than the proton transfer ratio 9 in the measurement should partially compensate for this. The z‐spectrum method uses eight fitted parameters, and is, therefore, probably over‐fitted. A full analysis of the interaction of the various parameters in the fit has not been performed, but while the chemical shift offsets are fairly well defined by the minima in the curve, the remaining curvature will include contributions from all relaxation rates as well as the exchange rate, reducing the expected accuracy in each of these. Given these limitations, the simple linewidth measurement may, therefore, be the most accurate. However, all values predict that at physiological pH spermine is unlikely to produce significant CEST signals at clinical field strengths. Because no significant CEST signals were seen in either spermine or citrate solutions at 11.7T and 310 K (Fig. 2), it is not expected that they would be seen in vivo even at field strengths available for preclinical studies.

## CONCLUSIONS

Citrate and spermine are glandular metabolites of interest for probing prostate diseases. Both contain labile ^1^H groups which in principle may create CEST contrast. Experiments at 11.7T and pH 6.5 found that the CEST effect was less than 0.2% mM^‐1^ for spermine, and was unmeasurable for citrate. Chemical exchange was determined to be in the fast and intermediate‐to‐fast exchange regimes for citrate and spermine respectively at 11.7T, under physiological conditions. At clinical field‐strengths such as 7T and 3T, these CEST effects would be further reduced due to coalescence of the exchanging ^1^H peaks with the bulk water peak. In vivo, these CEST effects are expected to be obscured by CEST from ^1^H in proteins and other metabolites.

## Supporting information


**Supporting Figure S1.** Structures of citrate (**a**) and spermine (**b**) showing protonation at physiologic pH, i.e., citrate has exchanging ^1^H‐O, expected to resonate just downfield of water (the hydroxyls in myo‐inositol resonate at +0.8 to +0.6 ppm relative to water 20 for example), and spermine has ten exchanging ^1^H nuclei which resonate at approximately 3.0 and 3.7 ppm relative to water (see Figure 1).
