# Supplementary material for: Determining the chemical exchange saturation transfer (CEST) behavior of citrate and spermine under in vivo conditions
Source: Magn Reson Med. 2015 Oct 15;76(3):742–6. doi: 10.1002/mrm.25997 (PMC5042183; doi:10.1002/mrm.25997)
Supplement: Supplementary file 1 — Supporting Figure S1. Structures of citrate (a) and spermine (b) showing protonation at physiologic pH, i.e., citrate has exchanging 1H‐O, expected to resonate just downfield of water (the hydroxyls in myo‐inositol resonate at +0.8 to +0.6 ppm relative to water 20 for example), and spermine has ten exchanging 1H nuclei which resonate at approximately 3.0 and 3.7 ppm relative to water (see Figure 1). [file MRM-76-742-s001.docx]

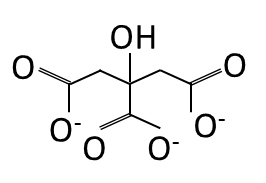

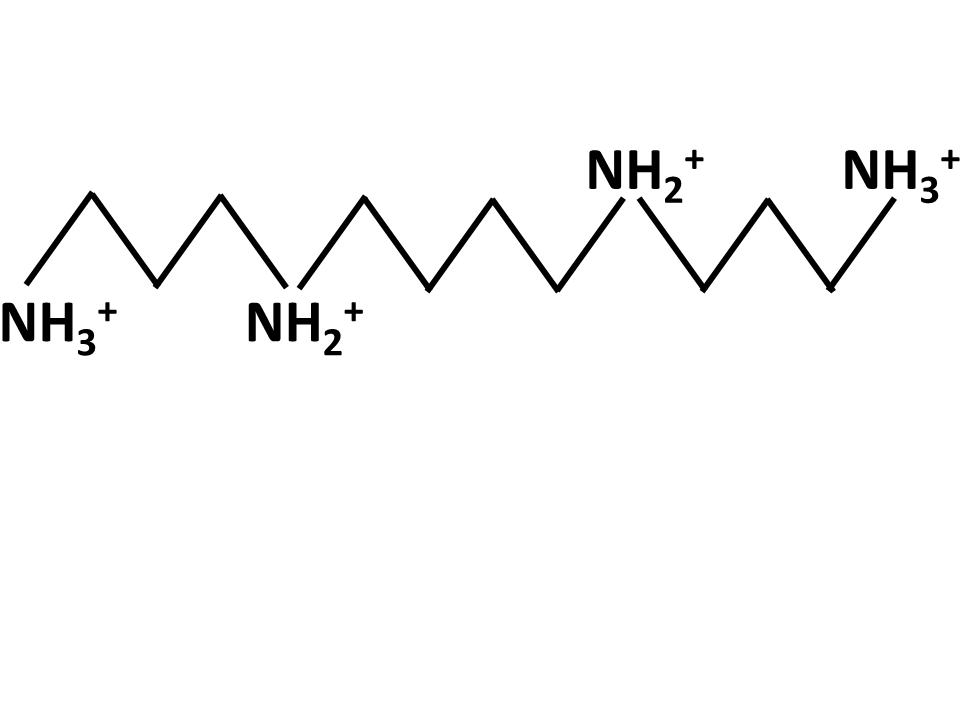


b)

a)

**Supporting Figure S1**

Structures of citrate (a) and spermine (b) showing protonation at physiologic pH *i.e.* citrate has exchanging ^1^H-O, expected to resonate just downfield of water (the hydroxyls in myo-inositol resonate at +0.8 to +0.6ppm relative to water (20) for example), and spermine has ten exchanging ^1^H nuclei which resonate at approximately 3.0 and 3.7 ppm relative to water (see Figure 1).
